# Bed-Bugs

**Published:** 1869-03

**Authors:** 


### BED-BUGS.

A LADY, at our elbow, who has never seen a bed-bug in her house says, she manages it in this wise:

Invariably, since she has been a housekeeper, her beds, whether of feather or hair, are entirely removed from the bedstead into an adjoining hall, once a week, and every crevice or button is thoroughly brushed out with a bristle brush; every crack and joint or other crevice in the bedstead is treated in the same way, and everything is thus left exposed for several hours to the light and air; now and then, say once a month, the slat-ends, slats and other harboring places are plentifully washed with clean cold water. Let every good housekeeper go and do likewise.

